# Supplementary material for: Patterns of Intron Gain and Loss in Fungi
Source: PLoS Biol. 2004 Nov 30;2(12):e422. doi: 10.1371/journal.pbio.0020422 (PMC532390; doi:10.1371/journal.pbio.0020422)
Supplement: Table S1 — Also available at http://genes.mit.edu/NielsenEtAl/. (4.3 MB ZIP). [file pbio.0020422.st001.zip › NielsenEtAl/html/122.html]

AN3451.1.NCU09297.1.MG03487.1.FG07854.1


```
 CLUSTAL W (1.82) Multiple Sequence Alignments - Introns Inserted


Sequence 1: NCU09297.1	1249 aa
Sequence 2: MG03487.1	1266 aa
Sequence 3: FG07854.1	1220 aa
Sequence 4: AN3451.1	1214 aa
Alignment Length: 1307 aa
Number Identitical Residues: 695 aa
Alignment Score (without introns) 32295


MG03487.1 	MDNTINFDINDALK-YLNDPASIPTPEADSALFDCENDPDSLT-NAVINPVLNPIVDAIA
NCU09297.1	MD-SINFDINNALKHYMDDPASIPTPEADSALVDCENEPESLGDNGLINSILNPIVDAVA
FG07854.1 	MD-TVDFDLNDALKHYMSDPASIATPEADGALFDCENDPDALT-LPVVNSVLNPIVDAVA
AN3451.1  	MEERIRFDINESLKYYLSDPNSVPTPEADTELLGFETEPEQLP-STVIDNVLNSIVDAVA
          	*:. : **:*::** *:.** *:.*****  *.. *.:*: *    ::: :**.****:*

MG03487.1 	ENPEAITRSAHFDSLQFLLK~CAPSCVLPIPYSHSSPTEARPARSRKRALQQRSPDNIGL
NCU09297.1	ENPDAITRAHIFDSLQFLLK2---------------------------------------
FG07854.1 	DNPDAIMRASNMDSLQFLLK~LAPISLHPSPDSPTSLG----------------------
AN3451.1  	ENPEALARASSFDSLQFLLK~CAFAPNPEPLVGPFGPN----------------------
          	:**:*: *:  :********  :         .  .                        

MG03487.1 	DSELFNHSRYTAFLPAHALSKIFDVITSGLAAETEAVAHDLES-DEQDLIAHHKQLLGIY
NCU09297.1	---------YTSFLSAHALSKIFDLITSGLAAEADVVHHDLEA-EEQELLAHHKQLLDIY
FG07854.1 	HNSELENPRYTAHLPTHALSKIFDLIMSGLSAEADSVHSDIDSPDEQDSVPHHKRLLEIY
AN3451.1  	-SELLNHYRYSNFLPTKSLSKLLDLIVSGLSVEADIIHGDLES-DEQDSIQPHKELLEMY
          	 ..  .   *: .*.:::***::*:* ***:.*:: :  *::: :**: :  **.** :*

MG03487.1 	SFLMQWTIACVETKAAEKSSAAPTARRGVKPKGKKAATAGAEDGNWDSAMQLQTALDTMC
NCU09297.1	AFLLQWTIAGVEAKAAEKASNTVTTRGRGKPK-----KPTGRDGTWDSSAQLETALNTMV
FG07854.1 	GFLLQWTIAAVETKAAEKTTTAPVARGRGKPK---KGSAKDKDAAWDSATQLQAALEVMC
AN3451.1  	GFILQWALSAVELKAAEKPAEALPARRGTGKS---GRPRTTKDSNWDGTAQIQVAMETMC
          	.*::**::: ** *****.: :  :*     .         .*. **.: *::.*::.* 

MG03487.1 	KVLRLKLAKIFLTTSERDTFIGLVCRPVYLILESEQRVKNTPIRMHAFKVLCMAVKHHGH
NCU09297.1	KALRLKLARIFMTTSERDTFIGLLTRPVYMILESEQRTKNTSIRMHCWKVLCVAIKHHGH
FG07854.1 	KVLKLKLSKIFLTTSERDTFIGLLTRPVYMVLESEQRVKTTAIRMHCFKVLCIAVKHHGH
AN3451.1  	KVMKLKLSKIFLTTSDRDTFINLFTRSSYLILESEQRVKSMAIRMHAFKVLCIAVKHHGH
          	*.::***::**:***:*****.*. *. *::******.*. .****.:****:*:*****

MG03487.1 	GYA1AQTAVVQNLTYFEHLSEPMAEFLQILSEQYDYEQLAAEIMV~ELSGKEFSSNDTKG
NCU09297.1	GYA~AQISIVQNLTYFEHLAEPMAEFLHILAEQYDYPQLAEEILR~ELSNKEFNSNDNKG
FG07854.1 	AYA1AQINIIQNLTYFEHLSEPMAEFLHILAETYDYPQLADEVLR~EISNKEFNSNDTRG
AN3451.1  	GFG1AQTSIVQSLTYFEHLSEPMAEFLHILAEQYDYPQLSDEILK2ELGNKEFNSNDTRG
          	.:. **  ::*.*******:*******:**:* *** **: *::  *:..***.***.:*

MG03487.1 	PKSVSTFITKLSELAPRLVIKQVPQLIKQMDSE0SYTLRCALIEVCGNMIAYLSRLDEKG
NCU09297.1	PKSVSTFMIKLSELAPRIIIKQVTLLAKQLDSE~SYTLRCALIEVFGNMLIHLSKSAERG
FG07854.1 	PKSVSSFIAKLSELAPRLVIKQMTMLAKQLDSE0SYTLRCALIEVCGNMVAYLSKQDERS
AN3451.1  	PKSVSAFIIKLSELAPRLIIKQMTLLAKQLDSE0SYTLRCAVIEVCGNLIADLSKQEERT
          	*****:*: ********::***:. * **:*** *******:*** **::  **:  *: 

MG03487.1 	ENHKTQLDAFFGILQERFLDVNPYCRCRTIQVYVRLCDLEHKFPKRRQKVAELACQSLED
NCU09297.1	ENHKTQMNAFFDVLEERFLDINPYCRVRAIQVYIKLCELEQKFPKRRQKAAELACRSLED
FG07854.1 	ENHKSQLNAFFDVLEERFLDINPYCRCRTLQVYMKLCDLAQKFPKRRQKAAELACRSLED
AN3451.1  	DNYKSQINAFFDVLEERFLDINPYCRCRAIQVYMRICDLEQKFPKRRQAAAELAARSLED
          	:*:*:*::***.:*:*****:***** *::***:::*:* :******* .****.:****

MG03487.1 	KSSNVRRNAIKLLGCLIKTHPFSAVHGAQLNRSEWQARLDQVDAELDALKPPVDAPGLD-
NCU09297.1	KSSHVRRNAIKLLGTLIRTHPFTALHGAQLARKDWQERLGKVEEELNALKPPVDAPGLDG
FG07854.1 	KSSNVRRNAIKLLGTFIKTHPFTVMHGAQLSRKEWQARLDKVEEELDALRPP-GVPGFGS
AN3451.1  	KSSNVRRNAIKLIAKLVSTHPFSVMHGGQLSLKEWTERLDAVDAELNALRPP-ETPGFDS
          	***:********:. :: ****:.:**.**  .:*  **. *: **:**:**  .**:..

MG03487.1 	-GGNTTVDTALLDDATQIESPQKQHPSQMSDEEKMAAIRKAQEEAATTEAINNLTLKKKY
NCU09297.1	EAGNTSVDPALLDDATQMMESPRKQPSEMTEEEKIAAIRRAQEEAATSEAIEKLTLTKRY
FG07854.1 	DQANTTVDNELLDEATQLASPQK--PTQMTDEDKVAAIQKAQEEAATGEAIEKLTLTRRY
AN3451.1  	-GDASHIDSELLDDATQLPDDSPSKAPRMSEEEKTIAMKKAAEQAATSELLTRLQLTRKY
          	    : :*  ***:***: .   . ...*::*:*  *:::* *:*** * : .* *.::*

MG03487.1 	YTEALRFISVIHEATGTICQLLGSKNKSEVIEAIDFFEIGDAYNIEQNKVGIRRMLRLIW
NCU09297.1	YSEALKFIDVLHEATETVCQLLGSKNKSEVIEAMDYFEIGDAYNIEQNKIGIRRMLRLIW
FG07854.1 	YNEAIKFIEVIDEATTIICQLLGSRNKSEVIEAIDFFEVGDAYNIEQNKVGIRRMLRLIW
AN3451.1  	YNEAIRFIEVLHAASNIVTQLLSSKNKSEAIEAMDFFVMLDAYKVETARTGIRRMLRLIW
          	*.**::**.*:. *:  : ***.*:****.***:*:* : ***::*  : **********

MG03487.1 	TKGNSDEGKGVQSHLIDCYKRLFFEAPGSFSPNDAANYIARNMISLTFGATPAELTSLEQ
NCU09297.1	TKGNSDEGKGVQTHLIECYKRLFFEAPGNFNANDAATYIARNMISLTFGATPAELTSLEQ
FG07854.1 	TKGNSDEGKGVQTHLIECYKRLFFEAPDSFSPNDAANYIARNMISLTFGATPAELTSLEQ
AN3451.1  	TKGNSDEGKGVQTHLIDCYKGLFFEAPDTFSPNDAANYIARNMLSLTFGATPAELTCLEQ
          	************:***:*** ******..*..****.******:************.***

MG03487.1 	LLATMMKGGMIHEIVIHKLWQVYGVQKREISRTQRRGAIIVLGMLATANPEIVVGEMEAM
NCU09297.1	LLSTMMKSGMISDLVIAKLWQVYGVQKREISRTQRRGAIIVLGMLATANPEIVVGEMETM
FG07854.1 	LLATMMKGGMIPEVVVNKLWQVYGVQKREISRTQRRGAIIVLGMLATANPEIVVGEMETM
AN3451.1  	LLSTMMRAGHISDAVIAKLWQVYGVQKKEISRTQRRGAIIVLGMIALADPEIVIKEIEIM
          	**:***:.* * : *: **********:****************:* *:****: *:* *

MG03487.1 	LRTGLGSLGRSDLQLAKFTCVALRRINPTGRQAKDSPVKFSRLPNDHAVLARLAAITDVP
NCU09297.1	LRTGLGAHGRADLQLAKYTCVALRRINPTGRQSKDSGIKFSRLPNDHALLIKLAAITEVP
FG07854.1 	LRTGLGAYGRSDLQLAKFTCIALRRINPTGRQAKDSTVKFSRLPNDHAVSVRLAAITEVQ
AN3451.1  	LRIGLGSLGRSDLILAKYTCIALRRMVP-GRQAKSKESGISKLTNDHPVLTKLAAMVETV
          	** ***: **:** ***:**:****: * ***:*..   :*:*.***.:  :***:.:. 

MG03487.1 	SDSKEWFGVAEQAINAIYAISKHPDVLCSEIIRHKTKQVFG--PRSRPSSRDE------N
NCU09297.1	TDSKEWYGVAEQAINAIYAIAKHPDVLCSEIIRRKTKGVFARPPKSRSASHEEKPSPTES
FG07854.1 	SDSKEWYGVAEQAISAIYAVSKHPDTLCSDLIRRKARQVFG---QSRSPPSSQ-----PS
AN3451.1  	SDSKEWYGVAEQAISAIYALSKHPDVLCSDIVKRKTRSVFQ--PQTRPPSSRS-------
          	:*****:*******.****::****.***:::::*:: **   .::*...  .       

MG03487.1 	AAPHPTQ---------SDPATADDAAESTP--AAPAKPKRDNAIELSQLLFIVGHVAIKQ
NCU09297.1	AEPTPAEEPNPDAMDEDEPTSAQAPVATQPEDAEAKKQHREHAIALSQLLFIVGHVAIKQ
FG07854.1 	SRPGSRDE--------TKPVPMEDQIQSQLQSQGEKHKKRDNAIALSQLLFIVGHVAIKQ
AN3451.1  	------------------PDNEDGQRPGTASTEKSVSGQKTSSAALSQLLFIVGHIAIKQ
          	                  *   :       .       ::  :  **********:****

MG03487.1 	IVHLEACELDFKRRKQEKEKSAAAAKEAEEADARRSSKARASTATAGGKEKSKEEPADEL
NCU09297.1	IVHLELCELDFKRRKQEKEKAAGTTSKRTSLSASASTNRRKS---GGPKAAKDEEEQDEL
FG07854.1 	IVHLELCELDFKRRKQEKEKATPAKND------------------------KDKEDADEL
AN3451.1  	IVHLELCELDFKRRKAEQEKNKPLNMAVQK--------------------NDEPGENDEL
          	***** ********* *:**         .                     ..    ***

MG03487.1 	DMIGGTTEDDFTEAMQHIRERELLYGPESLLALFGPLVSEICANNTTYRNRGLQAAATLC
NCU09297.1	DLIGGTTEDDFTEAIFHIRERELLYGPQSLLAMFGPMVSEICANNTTYRNRELQQAATLC
FG07854.1 	DLIGGTTEDDFTEAMAHIRERELLYGPSSLLAVFGPLVSDICANNTTYADKGLQAAATLC
AN3451.1  	DLIGGTTEDDFTEAIAHIRERELLYGANSLLSNFGPLVAEICANNNTYSDRNLQAAATIC
          	*:************: **********..***: ***:*::*****.** :: ** ***:*

MG03487.1 	LAKLMCVSAEYCEANLPLLITIMERSSDATVRSNAVIALGDMAVCFNHLIDENTDFLYRR
NCU09297.1	LAKLMCVSSEYCEANLPLLITIMERSVDPTVRSNAVIALGDMAVCFNHLIDENTDFLYRR
FG07854.1 	LAKLMCVSAEYCETNLPLLITIMERSPNATVRSNAVIALGDMAVCFNHLIDENTDFLYRR
AN3451.1  	MAKLMCVSAEYCEKNLPLLITIMERSEDPIVRSNAVIALGDMAVCFNHLIDENTDFLYRR
          	:*******:**** ************ :. ******************************

MG03487.1 	LADPEPSVKRTCLMTLTFLILAGQVKVKGQLGEMAKCLEDEDKRIADLARMFFTELSTKD
NCU09297.1	LSDPDPSVKRTCLMTLTFLILAGQVKVKGQLASMALCLEDGDKRIADLARMFFTELSTKD
FG07854.1 	LADDDASVKRTCLMTLTFLILAGQVKVKGQLGEMAKCLEDDDRRIADLARMFFTELSTKD
AN3451.1  	LNDDDASVKRTCLMTLTFLILAGQVKVKGQLGEMAKCLEDDDKRIADLSRMFFTELAGKD
          	* * :.*************************..** **** *:*****:*******: **

MG03487.1 	NAVYNHFVDMFSLLSAETALDEESFRRIIKFLLGFVEK0DKHAKQLAEKLAARLARCDTE
NCU09297.1	NAVYNHFVDMFSLLSSDPSLEEEGFRRVVKFLLGFVEK~DRHAKQLAEKLAARLQRCDTE
FG07854.1 	NAVYNHFVDMFSLLSAGDNMDEDSFRRIIKFLLGFVEK0DKHAKQLADKLAARLGRCETE
AN3451.1  	NAVYNHFVDMFSLLSAERNLEEGALRRIVKFLIGFIEK0EKHARQLAEKLAARLPRCETE
          	***************:   ::* .:**::***:**:** ::**:***:****** **:**

MG03487.1 	RQWNDVAFALGLLQHKNEEIAKQVSEGFKLIQSSA-----------------
NCU09297.1	RQWNDVAFALGLLQHKNEEIAKVVSEGFKVVPSGQQGMQATQRATQATQARA
FG07854.1 	RQWNDVAYALGILQHKNEEITKLVSEGYRVVHSSA-----------------
AN3451.1  	RQWNDVAYTLSLLPHKNEEIAKIVAGGFNKVINMATA---------------
          	*******::*.:* ******:* *: *:. : .   .
```
